# Malignant Phyllodes Tumor of the Breast with Metastasis to the Pancreas: A Case Report and Review of Literature

**DOI:** 10.1155/2018/6491675

**Published:** 2018-06-27

**Authors:** R. A. Amir, Rola S. Rabah, S. S. Sheikh

**Affiliations:** ^1^Imam Abdulrahman Bin Faisal University, Dhahran, Saudi Arabia; ^2^Pathology Services Division, Johns Hopkins Aramco Healthcare, Dhahran, Saudi Arabia

## Abstract

Phyllodes tumor (PT) is a rare tumor of the breast accounting for approximately 1% of all breast neoplasms. In 1838, J. Muller coined the term “cystosarcoma phyllodes” based on the leaf-like projections of the tumor extending into the cystic spaces and sarcomatous stromal growth. However, seeing as up to 70% of phyllodes tumors are benign, “cystosarcoma” was removed, and the tumor is now recognized simply as phyllodes tumor. It is mainly seen in females between the ages of 35 and 55. Although most phyllodes tumors are benign, malignant cases do uncommonly occur, 22% of which have distant metastasis typically to the lungs and bones. Rarely, this tumor metastasizes to other locations. Herein, we report a case of malignant phyllodes tumor with metastasis to the pancreas. According to our knowledge, only 3 case reports of pancreatic metastasis from malignant phyllodes tumor have been reported in literature thus far. We aim to increase awareness among physicians of this rare metastasic potential of the uncommonly encountered malignant phyllodes tumor.

## 1. Introduction

Phyllodes tumor is a rare tumor of the breast comprising about 1% of all breast tumors. In 1838, Johannes Muller created the term cystosarcoma phyllodes based on the leaf-like projections extending into cystic spaces and sarcoma-like stroma of the tumor. However, seeing as up to 70% of phyllodes tumors are benign, “cystosarcoma” was removed, and the tumor is now recognized simply as phyllodes tumor. This tumor is mainly seen in females between 35 and 55 years of age. Although the vast majority of phyllodes tumors behave in a benign fashion, there are cases in which these tumors are malignant and have even metastasized. Usually, PTs metastasize to the lungs and bones, rarely will they metastasize to other locations. Herein, we report a case of malignant phyllodes tumor with metastasis to the pancreas. This, according to our knowledge, has only been previously reported in 3 case reports in literature.

## 2. Case Presentation

**Figure 1:**
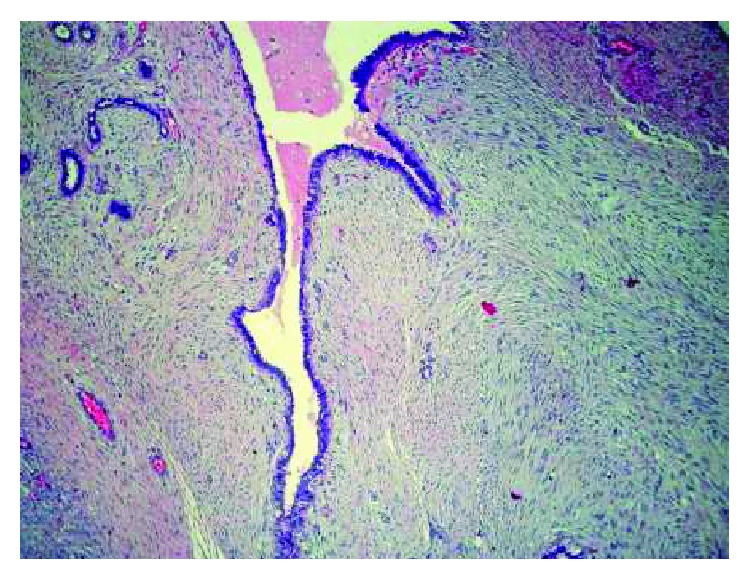
Prominent leaf-like pattern of the ducts surrounded by stromal overgrowth.

**Figure 2:**
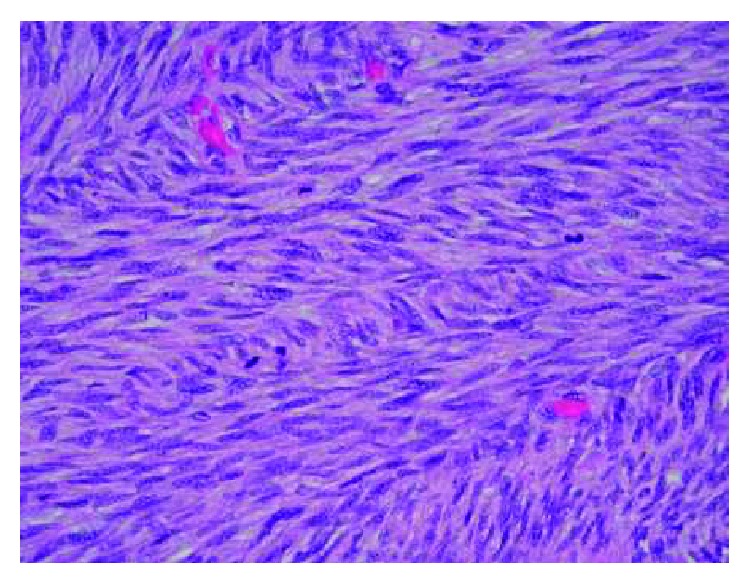
Hypercellular stroma with herringbone pattern, marked atypia, increased mitosis, and complete absence of ducts.

**Figure 3:**
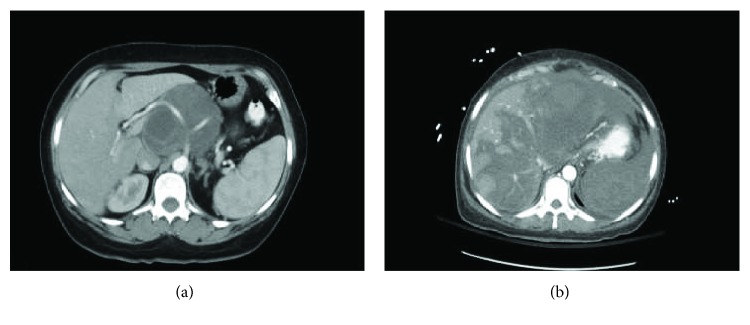
Abdominal CT scan showing (a) pancreatic mass involving the head of the pancreas, surrounding the celiac artery, in contact with the anterior aspect of the inferior vena cava, and (b) greatly enlarged pancreatic mass, extravasation of contrast from proximal splenic artery, and multiple heterogenous enhancements of the liver most likely indicating liver infarcts.

**Figure 4:**
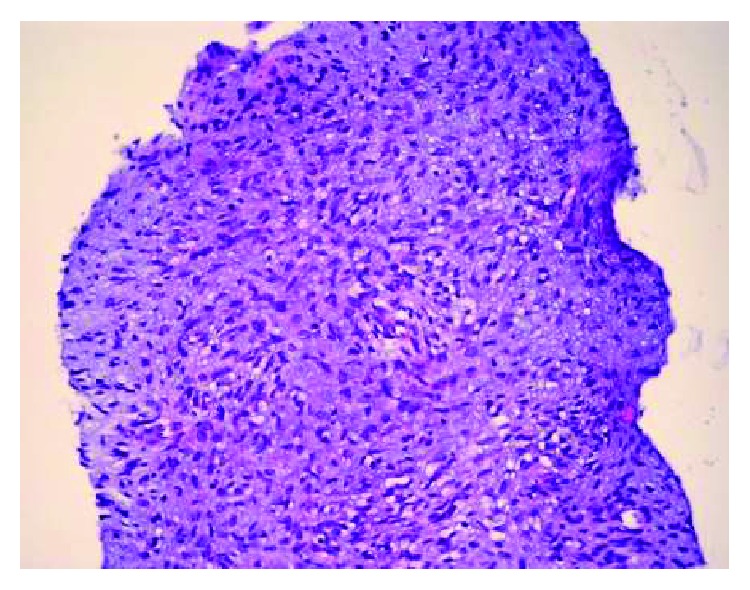
Spindle cell proliferation with focal herringbone pattern, cellular atypia, and increased mitosis.

**Figure 5:**
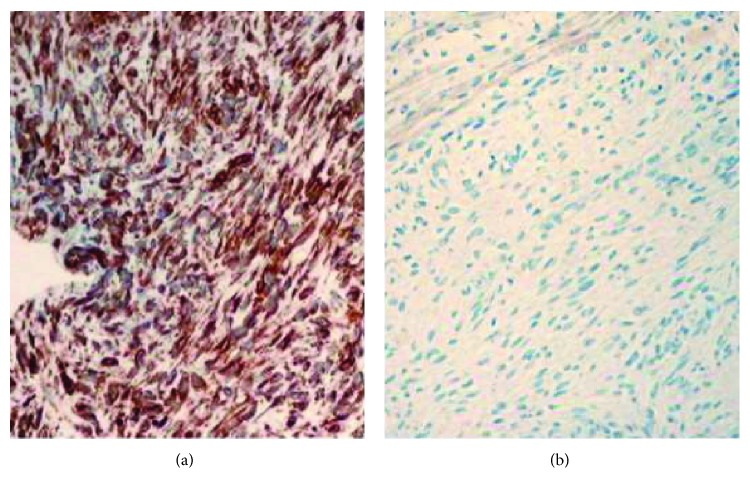
Immunohistochemistry staining showing (a) neoplastic cells strongly positive for vimentin and (b) malignant cells negative for cytokeratin, actin, and s100.

A 34-year-old female who has no previous clinical illness presented in 2000 with a large irregular mass, estimated clinically to be around 5 × 6 cm by physical examination, involving the right breast while she was lactating. There were no other signs or symptoms. FNA was done to rule out breast cancer, and the specimen showed few foamy macrophages and rare clusters of ductal cells, with focal secretory lactational changes. Based on that, it was determined that the specimen was negative for malignancy. The tumor continued to grow, and in June 2004, FNA of the right breast was repeated and showed large staghorn-shaped sheets of uniform ductal cells with no cytologic atypia along with myoepithelial cells. There were fragments of fibrotic stroma and bare nuclei scattered in a bloody background, and so, a diagnosis of fibroadenoma was made. Two months later, the patient underwent excision of the tumor in another healthcare facility. Examination of the slides showed an overgrowth of epithelial and stromal components in pericanalicular and intracanalicular patterns with irregular large cystic spaces showing protruding leaf-like structures (Figure 1). The stromal cells showed plump elongated slightly dysmorphic nuclei with occasional prominent nucleoli. Other areas showed significant cellular growth of the stromal spindle cells which appeared to be arranged in long intersecting fascicles and growing in herringbone pattern (Figure 2). Within the spindle cell growth, extracellular mucin was noted. One focus showed an exclusive spindle cell growth which was moderately atypical with plump elongated hyperchromatic nuclei. Numerous mitosis was found, up to 20 in 10 high power fields in this focus. Entrapped epithelial ductal elements were occasionally seen in other areas exhibiting ductal epithelial hyperplasia. These features were consistent with malignant phyllodes tumor with stromal fibrosarcomatous overgrowth. The patient subsequently underwent mastectomy as the margins were focally involved. During the procedure, a mass measuring around 3-4 cm was found in the most posterior aspect of the breast and there was some indication that it may have invaded the pectoralis major muscle. All the breast tissue, the tumor, and some of the muscle fibers were removed. On gross examination, there were multiple tumor masses in the inner lower and outer upper quadrants, the largest being 3 cm. On microscopy, there was proliferation of atypical spindle and elongated plump cells with pleomorphic nuclei and occasional prominent nucleoli. Extracellular mucin was also identified. The atypical stromal cells formed herringbone fascicular growth pattern reminiscent to fibrosarcoma and numerous mitosis averaging around 14 mitosis in 10 high power fields. No residual phyllodes tumor elements were identified. The tumor nodules had a well-demarcated margin with focal infiltration of surrounding breast and adipose tissue. No lymphovascular invasion, necrosis, or heterologous differentiation was seen. All surgical margins and muscle fibers were not involved by the tumor. The skin and nipple did not show any involvement either. All these histological features were identical to those identified in her previous lumpectomy except that there was more pronounced atypia and no residual epithelial component of phyllodes tumor identified. No lymph nodes were identified. A year later, the patient began to suffer from acute bouts of pancreatitis and was admitted several times for this. In March of 2006, abdominal CT was performed and showed a heterogenous low attenuation soft tissue mass involving the head and body of the pancreas and was extending upwards. The celiac vessels and its branches were going through this mass but did not show any significant narrowing. There was obvious atrophy of the pancreatic tail and dilatation of the pancreatic duct. The mass was in contact with the anterior aspect of the inferior vena cava (IVC) with no clear fat plane in between (Figure 3(a)). Biopsy of the pancreatic mass showed uniform proliferation of elongated spindly cells which had coarse chromatin and mild to moderate nuclear pleomorphism. Some cells had plump hyperchromatic nuclei. Numerous mitotic figures were identified (Figure 4). The background showed variable amounts of collagen and stroma with focal areas of myxoid appearance. Immunohistochemical staining showed strong positivity for vimentin; however, the cells were negative for actin, S100, and cytokeratin (Figure 5). These findings were consistent with metastatic fibrosarcoma of the pancreas secondary to her primary breast lesion. Due to the location, extent, and nature of the condition, the case was deemed unresectable. Chemotherapy was initiated; and in the little chance that the tumor shrunk enough, the possibility of resecting the metastasis would be entertained. In January 2007, the patient presented to the ER with severe epigastric pain, where she collapsed, was hypotensive, and subsequently admitted. On examination, a tender mass in the epigastric area was felt. Urgent CT of the abdomen and pelvis with and without contrast showed a pseudoaneurysm in the splenic artery measuring around 3.5 cm. There was also expansion of the retroperitoneal mass and blood in the intraperitoneal cavity. There was heterogenous enhancement in the liver most probably indicating liver infarct. In addition, there were areas of the IVC which had markedly thinned wall and areas suspicious for active bleeding, especially from the proximal splenic artery (Figure 3(b)). The pancreatic fibrosarcoma had grown and eroded branches of the celiac artery with bleeding pseudoaneurysm along with infarct of the liver, spleen, and adjacent organs. The patient continued to be hypotensive with abdominal distention due to severe intra-abdominal bleeding and developed multiorgan failure and hemorrhagic shock leading to her death.

## 3. Discussion

PTs are benign breast tumors found predominantly in females between the ages 35 and 55 (median 45). The incidence is higher in Caucasians and is highest in Latino women [1]. Few cases of PTs have been reported during pregnancy. In general, these tumors are unilateral and unifocal, but can occasionally present as bilateral and multifocal. In the case series done by Barrio et al., only 3.4% of the 293 cases studied were bilateral PTs while the majority was unilateral [2].

Clinically, the tumor often presents as a well-circumscribed oval painless mobile lump in the breast. It has a biphasic growth pattern: the initial phase of slow growth followed by the second phase of rapidly accelerating growth over a period of weeks to months. It may be associated with changes of overlying skin including atrophy, skin thinning, bloody nipple discharge, or nipple retraction if the areolar region is involved. The mean size of PTs is around 4-5 cm, but cases of PTs up to 40 cm in size have been reported in literature [3]. The larger the tumor the more likely it is to be malignant.

On mammography and ultrasound, PTs lack distinguishing characteristics, and, thus, findings are not specific [4]. Preoperative diagnosis of PTs is extremely difficult as the only imaging findings suggestive of PTs are the rapid growth rate and/or very large size of what looks like a fibroadenoma. On mammography, PTs are seen as well-circumscribed round or oval-shaped lobulated masses which on occasion could contain some calcifications [5, 6]. On sonography, they appear as well-defined solid masses with heterogenous internal echos, with no posterior acoustic attenuation [7]. If a solid mass containing clefts or elongated fluid-filled spaces is seen on sonography, PT should be considered. With the abovementioned imaging techniques, it is difficult to distinguish a fibroadenoma from a PT, and it is impossible to differentiate a benign from a malignant PT. The full extent of the tumor and metastatic sites can be delineated using magnetic resonance imaging prior to surgical excision.

Histologically, PTs are biphasic fibroepithelial tumors consisting of epithelial and stromal elements. The epithelial ducts are organized in cleft-like and cystic structures surrounded by stroma arranged in leaf-like pattern. The stromal component has various histological appearances. Generally speaking, benign PTs' stroma shows regular fusiform fibroblasts, while the stroma of malignant PTs usually shows high cellular atypia, increased stromal cellularity, and increased mitotic count. On occasion, degenerative changes can be seen including bleeding, cystic degeneration, and necrosis [8].

Since 2003, PTs have been classified by the WHO into three main categories: benign, borderline, and malignant, based on various histological findings. These features include margin characteristics (permeative versus circumscribed), mitotic activity, degree of cellular atypia, stromal cellularity, and stromal overgrowth (proliferation of stroma in relation to the glandular component with the presence of stroma and no epithelium in at least one 40 power fields) [9]. Benign PTs are well circumscribed with an increase in stromal cellularity, mild to moderate atypia, mitosis < 4/10 hpfs, and no signs of stromal overgrowth. Borderline PTs have an even larger increase in stromal cellularity and atypia, mitosis of 4–9/10 hpfs, but no stromal overgrowth. Malignant PTs on the other hand present with permeative margins, significant increase in both stromal cellularity and atypia, mitotic count of 10/10 hpfs, and signs of stromal overgrowth as opposed to the previous two categories [10]. In a review of 19 series of PTs, 35–85% were categorized as benign, 7–40% borderline, and 7–45% malignant showing variable incidence reporting [11].

Metastasis of the PTs is seen in 5–20% of all patients with PTs [12]. The timing of metastasis appearance has been studied in multiple publication and ranged from as little as 1 month after primary therapy to as long as a decade posttreatment with an average of around 15 to 26 months. Most patients with metastasis die within 3 years of primary diagnosis [13]. It is important to note that a defining factor in the occurrence of metastasis is the primary tumor size [14].

Malignant PTs tend to metastasize hematogenously with lymph node metastasis seen only rarely. However, in the cases of malignant PT, axillary lymph nodes must be dissected. The main site of metastasis is the lungs (66–84.5%) followed by bones (28–39%) and less commonly the liver and brain. Other reported sites of metastasis include the skin, nasal and oral cavity, salivary gland, larynx, thyroid gland, pleura, heart, kidneys, adrenal glands, stomach, small and large intestine, spleen, and pancreas.

In our case, the patient had metastasis only to the pancreas. Metastasis of PT to the pancreas is uncommon with frequency of secondary pancreatic tumors varying between 3% and 12% of all pancreatic malignancies. Clinical symptoms of pancreatic metastasis are similar to primary pancreatic tumors. Often the patient presents with jaundice, abdominal or back pain, weight loss, poor appetite, nausea, vomiting, pale greasy stool, hypercoagulability (e.g., DVT), abnormalities and unevenness of fatty tissue due to fat necrosis, or diabetes. The most common primary tumors to give rise to metastasis to pancreas are carcinoma of lung, breast, gastrointestinal tract, prostate, and kidney. Less commonly metastasis from soft tissue sarcomas to the pancreas has been reported. As for phyllodes tumor metastasizing to the pancreas, there are only three previous case reports to date in literature indicating its rarity.
